# The Balanced Cross-Layer Design Routing Algorithm in Wireless Sensor Networks Using Fuzzy Logic

**DOI:** 10.3390/s150819541

**Published:** 2015-08-10

**Authors:** Ning Li, José-Fernán Martínez, Vicente Hernández Díaz

**Affiliations:** Centro de Investigación en Tecnologías Software y Sistemal Multimedia para la Sostenibilidad (CITSEM), Campus Sur Universidad Ploitécnica de Madrid (UPM), Madrid 28031, Spain; E-Mails: jfmartin@diatel.upm.es (J.-F.M.); vhernandez@diatel.upm.es (V.H.D.)

**Keywords:** cross-layer design, routing algorithm, fuzzy logic, balanced performance, dynamic weight

## Abstract

Recently, the cross-layer design for the wireless sensor network communication protocol has become more and more important and popular. Considering the disadvantages of the traditional cross-layer routing algorithms, in this paper we propose a new fuzzy logic-based routing algorithm, named the Balanced Cross-layer Fuzzy Logic (BCFL) routing algorithm. In BCFL, we use the cross-layer parameters’ dispersion as the fuzzy logic inference system inputs. Moreover, we give each cross-layer parameter a dynamic weight according the value of the dispersion. For getting a balanced solution, the parameter whose dispersion is large will have small weight, and *vice versa*. In order to compare it with the traditional cross-layer routing algorithms, BCFL is evaluated through extensive simulations. The simulation results show that the new routing algorithm can handle the multiple constraints without increasing the complexity of the algorithm and can achieve the most balanced performance on selecting the next hop relay node. Moreover, the Balanced Cross-layer Fuzzy Logic routing algorithm can adapt to the dynamic changing of the network conditions and topology effectively.

## 1. Introduction

In recent years, the wireless sensor network routing protocol has been studied in widely. Many excellent routing protocols have been proposed, such as AODV [1], DSR [2], DSDV [3],* etc.* However, as the research moves along, more and more researchers realize that the traditional Open System Interconnect Reference Model (OSI) cannot meet the current quality of service (QoS) requirements of a wireless sensor network; even the OSI model simplifies the network design and improves the robustness. The reasons for this issue are, firstly, the wireless sensor network is a serious resource-constrained network [4], e.g., the limited bandwidth, the limited energy, the limited data processing capability, *etc.* Secondly, the OSI model only allows the communication between the adjacent layers, which cannot adapt to the dynamic changing of the wireless environment flexibly. Recently, the research proposes a cross-layer method for wireless sensor network communication protocol design, and many cross-layer methods have been proposed [5,6].

The cross-layer design allows the different layers to communicate with each other to coordinate the resource allocation between different layers, and allows the routing protocols to adapt to the wireless environment and the various applications. The core idea of the cross-layer design is to achieve the optimal resource allocation automatically in different layers. The cross-layer design is flexible due to its adaptive ability to adjust to meet the QoS requirements and the network constraints at the same time. Among various different developed policies, two important methods are the optimization-based routing algorithm and the fuzzy logic-based routing algorithm. For the optimization-based routing algorithm, e.g., in [7], the authors propose the Delay-Aware cross-layer design for network utility maximization in multi-hop networks; in [8], the authors introduce a new cross-layer design framework which join the scheduling and power control together; in [9], the authors show the optimization method in a cross-layer design of the wireless network; in [10], the authors present a survey of cross-layer solutions based on the nature of the adaptation using a systematic evaluation of existing approaches and identify critical criteria applicable to generic cross-layer framework design. In [11,12,13,14], the authors research the layering as optimization decomposition, in detail, including the problems, the current status, the open issues, the mathematics, the network architectures, and the framework; more recent works can be seen in [15,16,17,18]. For the fuzzy logic-based routing algorithm, e.g., in [19], the authors take the remaining battery reserve capacity, the link quality, and transmission power into consideration, by using the fuzzy logic inference system to determine the next hop relay node. In [20], the authors use the number of link breaks, the interface queue length, and the type of application as the inputs of the fuzzy logic system to enable each mobile node to separately switch between a reactive routing mode and a proactive routing mode. In [21], the authors use a fuzzy logic system to coordinate the physical layer, data-link layer, and application layer for cross-layer design; the ground speed, average delay, and packets successful transmission ratio are selected as antecedents for the fuzzy logic system. More related works can be found in [22,23,24,25,26].

However, the recent works also have some disadvantages. First, both of the two types of routing algorithms mentioned above will become extremely complex when the number of constraints increases, which is unacceptable in a wireless sensor network as the computation capability of a node is limited. Second, due to the complexity, they have limited ability to deal with the issue of multiple constraints. Third, they cannot adapt to the dynamic changing of the network conditions,* i.e.*, when the constraint or the topology changes, the routing algorithm needs to be redesigned [27], which is not flexible in the mobile wireless network. Fourth, all these two types of routing algorithms are concentrated on the specific aspect of the network performance (such as the congestion, the energy consumption, or the throughput), so they cannot achieve the balanced performance of the network. Motivated by this, in this paper, we propose a new fuzzy logic-based routing algorithm, named the Balanced Cross-layer Fuzzy Logic (BCFL) routing algorithm. 

To the best of our knowledge, BCFL is the first that introduces the dispersion into the fuzzy logic-based routing algorithm instead of the absolute cross-layer parameter values as the fuzzy logic inference system inputs (which is different with the traditional fuzzy logic-based routing algorithm, such as the routing algorithms discussed in [19,20,21], *etc.*), the first in setting every cross-layer parameter a dynamic weight, and also the first that proposes the dispersion formula. Based on these innovations, the BCFL will achieve better performance than the traditional routing algorithms mentioned above. We learn the properties of BCFL and compare the performance of BCFL with the traditional fuzzy logic-based routing algorithm (the algorithm used in [19,20]) and the optimization-based routing algorithm (the algorithm used in [9]). The simulation results also show that the BCFL has better performance on selecting the next hop relay node than the other two algorithms. Moreover, the complexity of BCFL is much less than the other two algorithms. Finally, we apply the BCFL in a multiple constraints scenario; the result shows that the BCFL can deal with the multiple constraints perfectly. The advantages of BCFL are:

- the number of if-then rules will keep constant when the number of cross layer parameters increase;
- the BCFL has the capability to handle multiple constraints without increasing the complexity of the algorithm;
- we give each parameter a dynamic weight, which can dynamically change according to the network conditions;
- as we use the dispersion of each cross-layer parameter as the input, when the network condition changes, the if-then rules will remain stable; therefore, we do not need redesign the if-then rules;
- in BCFL, the large dispersion parameter has a small weight and the small dispersion parameter has a large weight, which can decrease the influence of the parameters whose dispersion are large and obtain a more balanced solution.

The rest of this paper assignment is as follows. In Section 2, we introduce the related works about the fuzzy logic-based cross layer routing algorithms. In Section 3, we will state the problem and introduce the notations and the definitions that we will use in this paper. In Section 4, we introduce the principle of the Balanced Cross-layer Fuzzy Logic routing algorithm. In Section 5, we compare the performance of the BCFL with the optimization-based algorithm and fuzzy logic-based routing algorithm, and talk about the performance in a multiple constraints scenario. In Section 6, we will discuss the conclusion and talk about the future development of BCFL.

## 2. Related Works

Due to the excellent properties of the fuzzy logic-based method, many fuzzy logic-based routing protocols have been proposed in recent years. In this section, we will introduce these routing algorithms briefly. The fuzzy logic-based method is effective in routing selection and cluster header selection, which is shown as follows.

### 2.1. The Fuzzy Logic-Based Algorithm Used in Routing Selection

In [19], the authors propose an energy-effective cross-layer routing protocol for wireless sensor networks based on fuzzy logic. In this protocol, for minimizing the consumed energy and maximizing the network lifetime, the algorithm takes the remaining battery reserve capacity, the link quality, and the transmission power of the neighbor nodes into consideration to select the next hop relay nodes, dynamically. In [20], for reducing the average end-to-end delay of the mobile *ad hoc* network, the authors propose a fuzzy logic-based adaptive cross-layer routing protocol for the delay-sensitive applications. In this algorithm, each node can switch between reactive routing mode and proactive routing mode based on the current node status separately. The algorithm uses the fuzzy logic controller to decide the routing model of each node. The inputs of the fuzzy logic controller are the number of link breaks, the interface queue length, and the type of application for each node. In [26], the authors introduce a new routing algorithm for the wireless sensor network to extend the network lifetime and balance the energy consumption by combining the fuzzy approach and the A-star algorithm together. In this algorithm, the remaining battery power, the number of hops to the destination node, and the traffic loads are taken into consideration to determine an optimal routing path from the source node to the destination node. In [28], to prolong the lifetime of the wireless sensor network, a fuzzy logic-based energy-optimization routing protocol is proposed. In this algorithm, the social welfare function is used to predict inequality of residual energy of neighbor nodes after selecting different next hop nodes. The algorithm computes the degree of energy balance based on the energy inequality. The fuzzy logic system uses the degree of node closeness to the shortest path, the degree of node closeness to sink, and the degree of energy balance to achieve the routing decision. Additionally, in [29], the node density, the delay, and the number of dead nodes are the inputs of the fuzzy logic system to select the next hop relay node to achieve the balanced energy consumption across all of the sensor nodes with minimum delay. More related works can also be found in [30,31,32,33].

### 2.2. The Fuzzy Logic-Based Algorithm Used in Cluster Head Selection

In [34], the author proposes an energy-aware distributed dynamic clustering protocol (ECPF). In ECPF, the non-probabilistic cluster head selection is implemented by introducing a delay inversely proportional to the residual energy of each node. Based on this, the cluster heads are selected according their remaining energy. The fuzzy logic is used to evaluate the fitness of a node to choose a final cluster head from the neighbor cluster heads. The inputs of the fuzzy logic system are the node degree and the node centrality. In [35], the authors propose a fuzzy logic-based energy-efficient multiple cluster head selection routing protocol for the wireless sensor networks. In this algorithm, the distance from the base station and the residual energy are the inputs of the fuzzy logic system to decide the possibility of the node to be the cluster head. In [25], considering the expected residual energy, the authors propose the fuzzy logic-based clustering approach with an extension to the energy predication to prolong the lifetime of the wireless sensor network. In this algorithm, the residual energy and the expected residual energy are the inputs of the fuzzy logic system, and the output is the chance of the node to be the cluster head. The new algorithm selects the cluster head considering the expected residual energy of the sensor nodes. More papers on the fuzzy logic-based cluster head selection algorithm can be found in [36,37,38,39].

## 3. Problem Statement

In this section, we will state the problem we need to solve in this paper and introduce the notations and the basic definitions that will be used throughout the paper.

Considering the disadvantages (which have been discussed in Section 1) in traditional cross-layer routing algorithms, for the new routing algorithm, the more cross-layer parameters we can get from other layers, the better the performance of the algorithm [6,40]. In addition, we should note the fact that highlighting one aspect of performance of the network may have impact on other aspects. Since the network constraints are not isolated, they can interact and influence each other. In the routing algorithm design, for getting a more balanced and effective solution, we should take as many cross-layer parameters into consideration as possible. To this issue, the new routing algorithm should have the ability to handle multiple constraints and, furthermore, not increase the complexity of the algorithm.

The main goal of this paper is to design a new routing algorithm which has the properties as follows: (1) the calculation will not seriously increase with the increased number of constraints; (2) the algorithm can handle as many cross-layer constraints as possible; (3) the algorithm should have the ability to adapt to the dynamic changing of the network conditions and topology, especially in the underwater wireless sensor network in which the network conditions and topology change frequently; and (4) the algorithm should achieve the balanced performance on selecting next hop relay node.

The notations and basic definitions that will be used throughout the paper are now introduced.

- n: The sequence number of different cross-layer parameter;
- i : The sequence number of different nodes;
- $\alpha_{n}$ : The weight of different cross-layer parameter;
- α : The weight matrix;
- $\beta_{n}$ : The cross-layer parameter matrix of parameter n;
- $\beta_{n}^{i}$ : The cross-layer parameter n of node i;
- $D_{n}$ : The dispersion of parameter $\beta_{n}$;
- D : The dispersion matrix; and
- $U^{i}$ : The utility of node i;

*Definition 1*: The weight matrix α is the set of different cross-layer parameter weights and can be expressed as:

(1) $$\alpha =\left[\alpha_{1},\alpha_{2},\alpha_{3},\cdots ,\alpha_{n}\right]$$

*Definition 2*: The cross-layer parameter matrix is the set of cross-layer parameters $\beta_{n}^{i}$ that are obtained from node i , and can be expressed as:

(2) $$\beta_{n}={\left[\beta_{n}^{1},\beta_{n}^{2},\beta_{n}^{3},\cdots ,\beta_{n}^{i}\right]}^{T}$$

*Definition 3*: The dispersion is used to decide the scatter of different cross-layer parameters; the dispersion matrix D; $\bar{\beta_{n}}$ is the mean of the cross-layer parameters from different nodes. $D_{n}$ and D can be expressed as:

(3) $$D_{n}=\frac{(\left|\beta_{n}^{1}-\bar{\beta_{n}}\right|+\left|\beta_{n}^{2}-\bar{\beta_{n}}\right|+\cdots +\left|\beta_{n}^{i}-\bar{\beta_{n}}\right|)/i}{\bar{\beta_{n}}}$$

(4) $$D={\left[D_{1},D_{2},D_{3},\cdots ,D_{n}\right]}^{T}$$

*Definition 4*: The utility of node i is used to decide the order of the neighbor nodes to be the next hop relay node, and can be expressed as:

(5) $$U^{i}=\alpha \times \beta_{n}$$

The object of BCFL is to calculate the value of $U^{i}$ and chose the most suitable next hop relay node according to $U^{i}$.

## 4. The Principle of the Balanced Cross-Layer Fuzzy Logic Routing Algorithm

**Figure 1:**
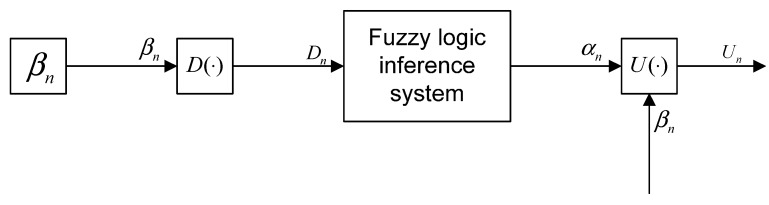
The principle of the BCFL.

In this section, we will introduce the principle of the Balanced Cross-layer Fuzzy Logic routing algorithm in detail. The principle of BCFL is shown in Figure 1. First, the source node collects the cross-layer parameters $\beta_{n}$ from its neighbor nodes and calculates the dispersion $D_{n}$ of each parameter. When the source node gets the dispersion $D_{n}$ , $D_{n}$ will be inputted into the fuzzy logic inference system to decide the weights $\alpha_{n}$. Finally, as shown in Equation (5), α and $\beta_{n}$ will be used to calculate the utility of each node, which is the reference to decide which neighbor node will be the best next hop relay node.

### 4.1. The Calculation of the Dispersion

When the source node wants to send packets, it will collect the cross-layer parameters from its neighbor nodes. After that, the source node will begin the pre-processing for the cross-layer parameters. The reason why the source node needs to do pre-processing to the cross-layer parameters is shown as follows.

As we know, the cross-layer parameters of different layers are variable,* i.e.*, the order-of-magnitudes are different. For instance, as discussed in Section 2, the distance to the base station is 1000 m, the number of link breaks is 5, the interface queue length is 20, the remaining battery power is 50%, the number of hops to the destination node is 10,* etc.* The orders of magnitude of these parameters are different; if we utilize these parameters directly, there will be some problems. Consider the parameters shown in Table 1.

**Table 1:** The raw data.

	Node 1	Node 2	Node 3
$\beta_{1}$	1000	2000	3000
$\beta_{2}$	0.8	0.5	0.1
$\beta_{3}$	27	49	15

In Table 1, we can find that the parameter $\beta_{1}$ is much larger than others. By means of the Balanced Cross-layer Fuzzy Logic routing algorithm, we can get the weights of each parameter that is shown in Table 1; the weights are shown in Table 2.

**Table 2:** The weight of the parameters.

	$\beta_{1}$	$\beta_{2}$	$\beta_{3}$
α	0.5	0.4	0.464

**Figure 2:**
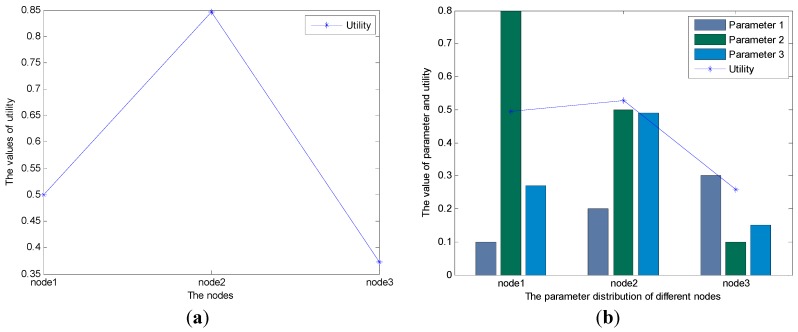
(**a**) The result of the traditional fuzzy logic-based algorithm (used in [19,20]) with the raw data; (**b**) The result of the new routing algorithm with the data in Table 2.

Considering Equation (5), for the data in Table 1, the utilities of node 1, node 2, and node 3 are *518.848*, *1022.936*, and *1507*, respectively. As a result, node 3 will be chosen as the next hop relay node. However, according the value of the parameters in Table 1, we can conclude that node 3 is not the best candidate as the next hop relay node. For proving this, we utilize the traditional fuzzy logic-based routing algorithm (used in [18,19]) to choose the best next hop relay node, with the result shown in Figure 2a. Figure 2a demonstrates that node 2 rather than node 3 is the best next hop relay node. The reason of this issue is that the parameters in Table 1 are not the same orders of magnitude, so the parameters whose orders of magnitude are much higher will have greater effects on the final result of BCFL. For addressing this problem, in the new algorithm, we transfer all the cross-layer parameters into the same orders of magnitude, which are shown in Table 3. The dispersions of the parameters in Table 2 are the same as that in Table 1 (this is also the reason why we use Equation (3) instead of the variance formula to calculate the parameters’ dispersion, because the variances in Table 1 are different with that in Table 2; the result of Equation (3) has nothing to do with the order of magnitude), thus the parameters’ weights are the same, too.

**Table 3:** The data after pre-processing.

	Node 1	Node 2	Node 3
$\beta_{1}$	0.1	0.2	0.3
$\beta_{2}$	0.8	0.5	0.1
$\beta_{3}$	0.27	0.49	0.15

Figure 2b shows the result of the BCFL that uses the parameters shown in Table 2. Figure 2b illustrates that node 2 will be the best next hop relay node, which is the same with the traditional fuzzy logic based routing algorithm shown in Figure 2a.

Therefore, we can conclude that the idea that setting all the cross-layer parameters into the same order of magnitude is an appropriate and effective method. In terms of this conclusion, more generally, we can use the numbers between 0 and 1 to represent the cross-layer parameters.

Once the pre-processing of the cross-layer parameters is finished, we can apply Equation (3) to calculate the parameter dispersion. Furthermore, the dispersions will be used as the fuzzy inference system inputs.

### 4.2. The Design of the Fuzzy Logic Inference System

**Figure 3:**
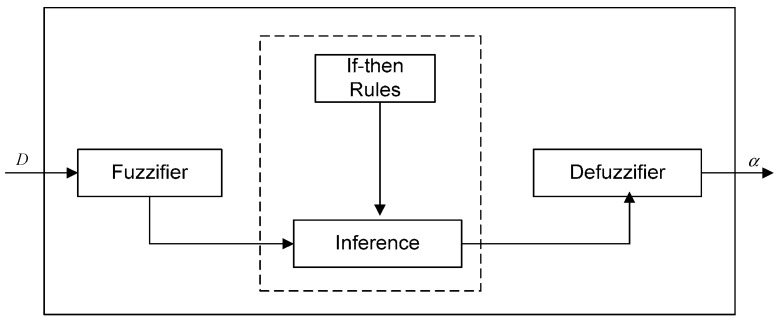
The principle of the fuzzy inference system.

**Figure 4:**
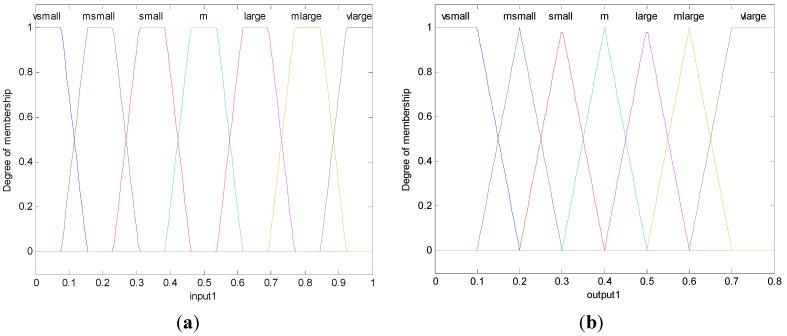
(**a**) The membership function of input; (**b**) The membership function of output.

Fuzzy logic has the advantages of easy implementation, robustness, and ability to approximate to any nonlinear mapping [41,42]. It is relatively simple to convert knowledge of domain experts to control algorithms. The design of a fuzzy-logic controller starts with constructing the membership functions for linguistic input/output and if-then rules. In BCFL, the input of the fuzzy logic inference system is the parameter dispersion and the output is the cross-layer parameter weight. The new fuzzy logic inference system is a single input-single output system, which is shown in Figure 3. In the rest of this section, we will introduce the membership functions and the if-then rules of BCFL in detail. The input and output membership functions are shown in Figure 4, and the if-then rules are shown in Table 4. The linguistic variables of input and output are: *very small (vsmall)*,* medium small (msmall)*,* small*, *medium*,* large*,* medium large (mlarge)*,* very large (vlarge)*, which are shown in Table 4.

As shown in Table 4, the large dispersion has small weight and the small dispersion has large weight. The reason is that the parameter whose dispersion is large will have a greater effect on the algorithm performance; however, in the BCFL, we intend to get the most balanced solution, so this situation should be avoided as much as possible. Therefore, in the new routing algorithm, the larger the dispersion is, the smaller value of $\alpha_{n}$ .

**Table 4:** The fuzzy if-then rules of BCFL.

**Input (** $D_{n}$**)**	Very small	Medium small	Small	Medium	Large	Medium large	Very large
**Output (** $\alpha_{n}$ **)**	Very large	Medium large	Large	Medium	Small	Medium small	Very small

From Figure 4 and Table 4, we can conclude that the if-then rules and membership functions will keep constant even when the network condition and topology change; moreover, the if-then rules are very simple in this algorithm. The BCFL is easy to design and is robust. On the contrary, in the traditional fuzzy logic-based routing algorithm (the algorithm used in [18,19]), the if-then rules will become unacceptable with the increasing influence of the parameters. In addition, when the network condition or the topology changes, the if-then rules need to be redesigned, which is not flexible.

Once inputting the parameters’ dispersion into the fuzzy logic system, the fuzzy logic inference will set the weights for each parameter based on the dispersion, which will be used to calculate the utility of each node.

### 4.3. The Calculation of the Utility of Each Node

The utility is used to decide the order of the neighbor nodes to be the next hop relay node. When we get the weights from the fuzzy logic inference system, we can use Equation (5) to calculate the utility of each node.

As defined in Section 2, the utility of node i can be expressed as:

(6) $$U^{i}=\alpha_{1}\beta_{1}^{i}+\alpha_{2}\beta_{2}^{i}+\alpha_{3}\beta_{3}^{i}+\cdots +\alpha_{n}\beta_{n}^{i}$$

where $\beta_{n}^{i}$ represents the cross-layer parameters (such as the distance to the base station, the number of link breaks, the interface queue length, the remaining battery power, the number of hops to the destination node,* etc.*) of node i ; $\alpha_{n}$ is the different parameter weight, which can be obtained from Sections 4.2.

The process of BCFL is shown as follows:
**Algorithm 1** The proposed fuzzy logic based routing algorithm (BCFL)
**Input:**
$\beta_{n}^{i}$
: The cross-layer parameters from node
i;
$\beta_{th}$
: The threshold of the cross-layer parameters;
**Output:**
 NR
: The rank of the neighbor node to be the next hop relay node;
**Function:**
 Dispersion(
$\beta_{n}^{i}$
): The function to calculate the dispersion of the cross-layer parameters;
 Fuzzylogic(
$D_{n}$
): The fuzzy logic inference function to calculate the weight of each cross-layer parameters;
 Utility(
$\beta_{n}^{i}$
,
$\alpha_{n}$
): The function to calculate the utility of each node;
 Probability(
$U_{n}$
): The function to decide the rank of the neighbor node to be the next hop relay node;
**Initialization:**
 Routediscovery=0;
   Nodestatus==false;
**Main:**
 While Routediscovery=1 do
    Broad hello message to neighbor nodes;
    For cross-layer parameters received from other nodes, store these parameters;
      if (
$\beta_{n}^{i}\geq \beta_{th}$
)
        Nodestatustrue←true;
      else if (
$\beta_{n}^{i}<\beta_{th}$
)
        Nodestatusfalse←false;
      end if
      while Nodestatus==true do
        $D_{n}$←Dispersion(
$\beta_{n}^{i}$
);
        $\alpha_{n}$←Fuzzylogic(
$D_{n}$
);
        $U_{n}$←Utility(
$\beta_{n}^{i}$,
$\alpha_{n}$
);
        NR←Probability(
$U_{n}$
);
      end while
 end while

## 5. Performance Evaluation

In this section, we will discuss the properties and the performance of the BCFL in detail. During the simulation in Section 5.1 and Section 5.2, generally, we do not specify the cross-layer parameters ( $\beta_{1}$ , $\beta_{2}$ , $\beta_{3}$… , $\beta_{n}$ ) to the specific factors (such as the distance to the base station, the number of link breaks, the interface queue length, the remaining battery power, the number of hops to the destination node, *etc.*); we use the random number sequence (from 0 to 1) to represent the value of the cross-layer factors (which has been proven in Section 4.1 that this method is appropriate and effective). In Section 5.1, we will compare the performance of the BCFL with the traditional fuzzy logic-based routing algorithm and the optimization-based routing algorithm; in Section 5.2, we will show the performance of the BCFL under multiple constraints.

### 5.1. The Performance of BCFL Compared with the Traditional Routing Algorithm

In this part, we compare the performance of the BCFL with the traditional fuzzy logic routing algorithm (used in [19,20]) and the optimization-based routing algorithm (used in [9]), respectively. In view of that the traditional fuzzy logic routing algorithm, the optimization-based routing algorithm has limited capability to handle multiple constraints, so in the simulation of this part, we only consider three constraints in the algorithm. As discussed in Section 4, we use the function *rand()* in MATLAB to generate the cross-layer parameters. The parameters are shown in Table 5.

**Table 5:** The cross-layer parameters.

	Node 1	Node 2	Node 3	Node 4	Node 5
$\beta_{1}$	0.4505	0.0838	0.229	0.9133	0.1524
$\beta_{2}$	0.602	0.263	0.6541	0.6892	0.7482
$\beta_{3}$	0.8258	0.5383	0.9961	0.0782	0.4427

**Figure 5:**
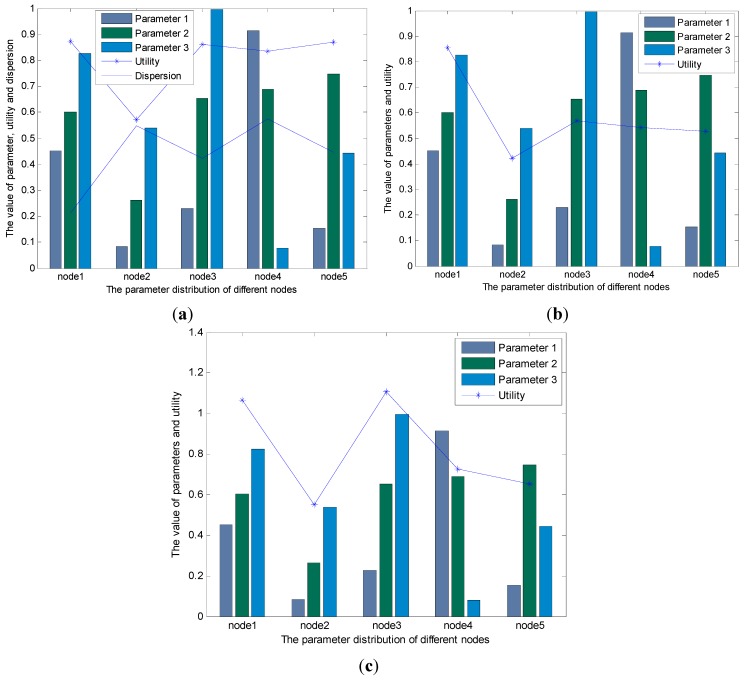
(**a**) The result of the BCFL; (**b**) The result of the traditional fuzzy logic-based routing algorithm (the algorithm used in [19,20]); (**c**) The result of the optimization-based routing algorithm (the algorithm used in [9]).

Figure 5a,b demonstrate that the new routing algorithm can come to the similar conclusion with the traditional fuzzy logic-based routing algorithm: the best next hop relay node is node 1; the performance of node 3, node 4, and node 5 are alike; and node 2 has the worst performance of these five nodes. The result also proves that the BCFL is effective and balanced. As shown in Figure 5a, the line graph of dispersion illustrates that the parameters in node 1 have the smallest dispersion; the dispersion of node 3 and node 5 are similar and all larger than node 1; node 2 and node 4 have the largest parameter dispersion. This line graph is consistent with the line graph of utility in Figure 5a (the node whose parameter dispersion is small will have large utility). For the utility in Figure 5a, node 1 is the largest, node 3 and node 5 are alike and they are the medium. Note that even node 2 and node 4 have the similar parameter dispersion, but since the parameters in node 2 are too small, then the utility of node 4 is greater than node 2. Therefore, the new routing algorithm can not only consider the balance but also the effectiveness of the network performance.

Note that the dispersion shown in Figure 5a is different with the dispersion that is expressed in *Definition 3*. The dispersion in *Definition 3* means the difference between the same cross-layer parameters from different nodes, e.g., the parameters $\beta_{1}^{1},\beta_{1}^{2},\beta_{1}^{3},\cdots ,\beta_{1}^{i}$ (the parameter 1 from node 1, node 2, …, node i). The dispersion shown in Figure 5a means the difference of different cross-layer parameters of the same node, e.g., the parameters $\beta_{1}^{1},\beta_{2}^{1},\beta_{3}^{1},\ldots ,\beta_{n}^{1}$ (the parameter 1, parameter 2,…, parameter n from node 1). Thus, the parameter dispersion shown in Figure 5a can represent the balance of nodes.

In Figure 5b, the utility of node 4 is larger than node 5; however, as shown in Figure 5a, the parameter dispersion of node 5 is smaller than node 4. Considering the parameter’s value and the parameter dispersion of these two nodes, we can conclude that node 5 is more appropriate as the next hop relay node than node 4. On this point, the Figure 5a,b prove that the BCFL can get a more balanced performance than the traditional fuzzy logic routing algorithm.

The Figure 5c shows the result of optimization-based routing algorithm. In Figure 5c, the node 3 has higher possibility to be the next hop relay node than node 1. However, as shown in Figure 5a,b, node 1 has a more balanced performance than node 3, thus node 1 is more suitable to be the next hop relay node than node 3. Furthermore, both in Figure 5b,c, the utility of node 4 is larger than node 5; but as discussed in Figure 5a, the node 5 has a more balanced performance than node 4. The reason for this issue is that in the optimization-based routing algorithm and traditional fuzzy logic-based routing algorithm, the large parameters will have a greater effect on the algorithm performance; and the nodes that these parameters belong to are easy to be chosen as the next hop relay node, which is consistent with the performance of node 4 and node 5 in Figure 5b,c, respectively.

**Figure 6:**
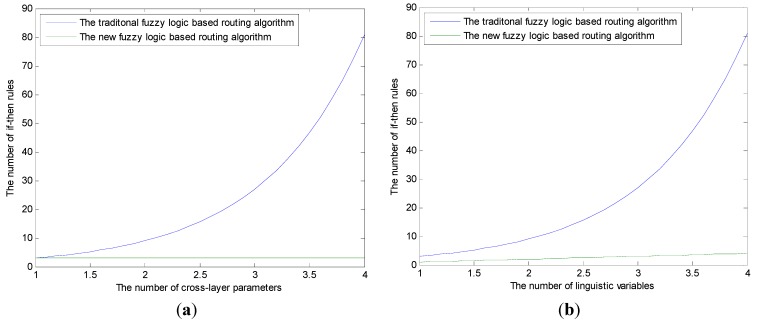
(**a**) The relationship of the number of if-then rules and the number of cross-layer parameters; (**b**) The relationship of the number of if-then rules and the number of the linguistic variables.

The Figure 5 indicates that the BCFL and the traditional fuzzy logic-based routing algorithm (used in [19,20]) have the similar performance, and they are all better than the optimization-based routing algorithm (used in [9]). On the other hand, even the traditional fuzzy logic-based routing algorithm has similar performance on selecting the next hop relay node with the BCFL, but considering the number of the fuzzy if-then rules, the BCFL will have greater advantage than the traditional fuzzy-based routing algorithm. As shown in Figure 6, in the traditional fuzzy-based routing algorithm, the number of if-then rules increases sharply with the increasing of the linguistic variables and the number of cross-layer parameters; moreover, this increase is exponential. On the contrary, in the BCFL, the number of fuzzy rules will stay constant when the cross-layer parameters increase (Figure 6a), and even when the linguistic variables increase, the growing of the fuzzy if-then rules is linear (Figure 6b), which is much less compared to the traditional fuzzy logic-based routing algorithm.

The Figure 6a,b demonstrate that the traditional fuzzy logic routing algorithm cannot handle multiple constraints. If the number of cross-layer parameters is large, the number of if-then rules will become too large to be acceptable. Moreover, the fuzzy rules have great effect on the fuzzy inference system performance, so an appropriate fuzzy rule is very important to the fuzzy logic system. For this point of view, the BCFL can reduce the complexity of the algorithm greatly; more importantly, the rules of the new algorithm are clear and fixed, which makes it easy to design and more accurate.

### 5.2. An Example Scenario for Multiple Constrains

The performance of the BCFL under multiple constraints will be shown in this section. From this scenario, we can find the greater advantage of the BCFL more directly.

In this simulation, we consider five nodes as the candidates of the next hop relay nodes and seven cross-layer parameters for each node. So the cross-layer parameter matrix is a 5 × 7 matrix. This matrix is generated by the function *rand()* in MATLAB. The parameters are shown in Table 6.

**Table 6:** The cross-layer parameters of multiple constraints.

	$\beta_{1}$	$\beta_{2}$	$\beta_{3}$	$\beta_{4}$	$\beta_{5}$	$\beta_{6}$	$\beta_{7}$
Node 1	0.0451	0.2238	0.2751	0.6273	0.571	0.8131	0.9861
Node 2	0.7232	0.3736	0.2486	0.0216	0.1769	0.3833	0.0300
Node 3	0.3474	0.0875	0.4516	0.9106	0.9574	0.6173	0.5357
Node 4	0.6606	0.6401	0.2277	0.8006	0.2653	0.5755	0.0871
Node 5	0.3839	0.1806	0.8044	0.7458	0.9246	0.5301	0.8021

**Figure 7:**
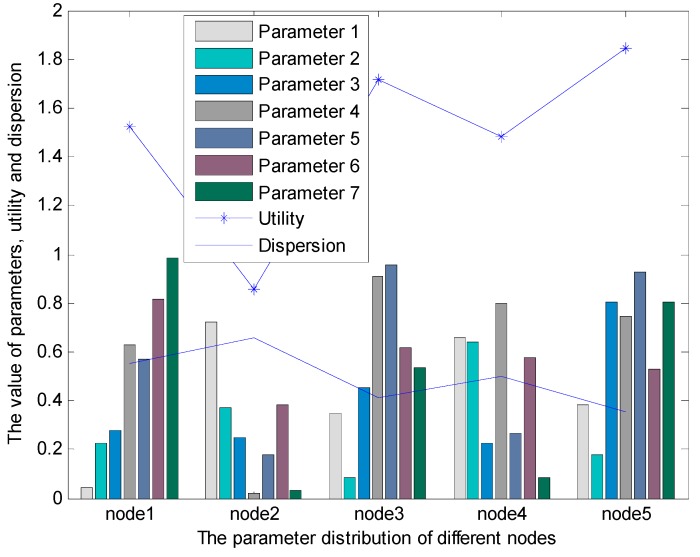
The result of the BCFL under multiple constraints.

The result of this scenario is shown in Figure 7.

From Figure 7, we can find that node 5 has the best performance among all the candidate nodes; as shown in Table 6 and the line graph of dispersion in Figure 7, the cross-layer parameters of node 5 are more balanced than the other nodes. From the line graph of utility in Figure 7, we can conclude that the performance of node 3 is better than node 1, node 1 has the better performance than node 4, and the performance of node 2 is the worst in these nodes. The simulation result is consistent with the character of the cross-layer parameters in different nodes, which is shown in the histogram and the line graph of dispersion in Figure 7.

The Table 6 indicates that the parameters of node 5 are not always the highest among all of the nodes, but they are the most balanced, which can be found in the line graph of dispersion in Figure 7. The line graph of dispersion demonstrates that the node whose parameters dispersion is small will have high probability to be the next hop relay node, and *vice versa*, because the small dispersion leads to the balanced performance.

In this scenario, as the number of cross-layer parameter is seven, so for the optimization based routing algorithm, the unknown variables and the functions are at least seven, then the calculation will be extremely complex; for the traditional fuzzy logic-based routing algorithm, when there are seven parameters in the system, the number of if-then rules is 2187 (assuming that the number of linguistic variables is three), which is unacceptable. However, for the BCFL, the number of if-then rules is constant and the same with the number of the linguistic variables (in BCFL, the number of if-then rules is seven), which is much simpler than the other two algorithms. This example also illustrates the huge advantage of the new fuzzy logic-based routing algorithm.

## 6. Conclusions

In this paper, we propose a new fuzzy logic-based routing algorithm (the BCFL routing algorithm). The innovations of this algorithm are: (1) we use the parameter dispersion rather than the absolute parameter value as the input of the fuzzy logic inference system, which can reduce the complexity of the algorithm observably; (2) the dispersion formula can guarantee that when the order of magnitude changes. the dispersion will remain unchanged; (3) the parameter whose dispersion is large will have small weight and whose dispersion is small will have large weight. According this, the BCFL can achieve some noble properties that the traditional routing algorithms cannot obtain:

- Simply, the if-then rules of the BCFL are very simple and they will stay constant with the increase in the constraints;
- Efficiently, the BCFL has excellent capability to deal with the multiple constraints without increasing the complexity;
- Adaptively, the BCFL can adapt to the changing of the network conditions and topology, especially in the underwater wireless sensor network in which the network conditions and topology change frequently;
- Balanced, the BCFL can get a more balanced solution than the traditional algorithms.

The BCFL is compared with the optimization-based routing algorithm (the algorithm used in [9]) and the traditional fuzzy logic-based routing algorithm (the algorithm used in [19,20]) (in Section 5.1). In addition, we also discuss the performance of the BCFL under multiple constraints (in Section 5.2). The simulation results in Section 5.1 show that the BCFL and the traditional fuzzy logic-based routing algorithm have similar performance, and they all better than the optimization-based routing algorithm. Moreover, even the performance of BCFL is similar with the traditional fuzzy logic-based routing algorithm, however, when considering the fuzzy rules, the BCFL has greater advantage than the traditional fuzzy logic-based routing algorithm used in [19,20]. In Section 5.2, the simulation results demonstrate the excellent capability of the BCFL on dealing with the multiple constrains, which is balanced, simple, and efficient.

However, in this paper, we propose the routing algorithm and evaluate the algorithm performance by simulation without a real scenario. Therefore, in the future, we will propose the routing protocol based on this algorithm and investigate the performance in a real scenario. In addition, the performance-related parameters of the routing protocol, such as the energy consumption, the time delay, and the memory use,* etc.* will be evaluated in future work. Moreover, how the routing protocol can work with the IEEE standards (such as the 802.15.4, 802.15.1) also deserves to be investigated. As the BCFL has remarkable performance on adapting to the dynamic changing of the network conditions and topology, in the future the BCFL can be used in the dynamic topology network, such as the mobile wireless sensor network, the *ad hoc* network, or the underwater wireless sensor network. Furthermore, this algorithm also can be used to select the cluster head in the cluster-based routing protocol.

## References

[1] Perkins C., Belding-Royer E., Das S. Ad-hoc On-Demand Distance Vector Routing. Proceedings of the Second IEEE Workshop on Mobile Computing System and Applications.

[2] David B.J., David A.M., Josh B. (2001). Ad Hoc Networking.

[3] Perkins C.E., Bhagwat P. (1994). Highly dynamic Destination-Sequenced Distance-Vector routing (DSDV) for mobile computers. ACM SIGCOMM Comput. Commun. Rev..

[4] Prathap U., Deepa S.P., Venugopal K.P., Patnaik L.M. Wireless Sensor Networks Applications and Routing Protocols: Survey and Research Challenges. Proceedings of the 2012 International Symposium on Cloud and Services Computing.

[5] Srivastava V., Motani M. (2005). Cross-layer design: A survey and the road ahead. IEEE Commun. Mag..

[6] Mendes L.D.P., Rodrigues J.J.P.C. (2011). A survey on cross-layer solutions for wireless sensor networks. J. Netw. Comput. Appl..

[7] Xiong H., Li R., Eryilmaz A., Ekici E. (2011). Delay-Aware Cross-Layer Design for Network Utility Maximization in Multi-Hop Networks. IEEE J. Sel. Areas Commun..

[8] ElBatt T., Ephremides A. (2004). Joint Scheduling and Power Control for Wireless Ad Hoc Networks. IEEE Trans. Wirel. Commun..

[9] Lin X., Shroff N.B., Srikant R. (2006). A Tutorial on Cross-layer Optimization in Wireless Networks. IEEE J. Sel. Areas Commun..

[10] Edirisinghe R., Zaslavsky A. (2014). Cross-Layer Contextual Interactions in Wireless Networks. IEEE Commun. Surv. Tutor..

[11] Chiang M., Low S.H., Calderbank A.R., Doyle J.C. Layering as Optimization Decomposition: Current Status and Open Issues. Processing of the Annual Conference on Information Sciences and Systems.

[12] Chiang M., Low S.H., Calderbank A.R., Doyle J.C. Layering as Optimization Decomposition: Questions and Answers. Processing of the IEEE Military Communications Conference.

[13] Chiang M., Low S.H., Calderbank A.R., Doyle J.C. (2007). Layering as Optimization Decomposition: A Mathematical Theory of Network Architectures. IEEE Proc..

[14] Chiang M., Low S.H., Calderbank A.R., Doyle J.C. Layering as Optimization Decomposition: Framework and Examples. Proceedings of the IEEE Information Theory Workshop.

[15] Shakkottai S., Shakkottai S.G., Srikant R. (2007). Network Optimization and Control.

[16] Georgiadis L., Neely M., Tassiulas L. (2006). Resource Allocation and Cross-Layer Control in Wireless Networks.

[17] Eryilamz A., Srikant R. (2006). Joint congestion control, routing and MAC for stability and fairness in wireless networks. IEEE J. Sel. Areas Commun..

[18] Lin X., Shroff N.B. Joint rate control and scheduling in multihop wireless networks. In Proceedings of the IEEE Conference on Decision and Control.

[19] Jaradat T., Benhaddou D., Balakrishnan M., Al-Fuqaha A. Energy Efficient Cross-Layer Routing Protocol in Wireless Sensor Networks Based on Fuzzy Logic. Proceedings of the 9th International Wireless Communication and Mobile Conference.

[20] Fathy C., El-Hadidi M.T., El-Nasr M.A. Fuzzy-based Adaptive Cross Layer Routing Protocol for Delay Sensitive Applications in MANET. Proceedings of the IEEE International Conference on Communications.

[21] Xia X., Ren Q., Liang Q. Cross-layer Design for Mobile Ad Hoc Networks: Energy, Throughput and Delay-Aware Approach. Proceedings of the IEEE Wireless Communications and Networking Conference.

[22] Rea S., Pesch D. Multi-Metric Routing Decisions for Ad Hoc Networks using Fuzzy Logic. Proceedings of the International Symposium on Wireless Communication Systems.

[23] Sonmez C., Isik S., Donmez M.Y., Incel O.D., Ersoy C. SUIT: A Cross Layer Image Transport Protocol with Fuzzy Logic Based Congestion Control for Wireless Multimedia Sensor Networks. Proceedings of the International Conference on New Technologies, Mobility and Security.

[24] Mhemed M., Aslam N., Phillips W., Comeau F. An Energy Efficient Fuzzy Logic Cluster Formation Protocol in Wireless Sensor Networks. Proceedings of the 3rd International Conference on Ambient System, Networks and Technologies.

[25] Lee J.S., Cheng W.L. (2012). Fuzzy-logic-based Clustering Approach for Wireless Sensor Networks Using Energy Predication. IEEE Sens. J..

[26] AlShawi I.S., Yan L., Pan W., Luo B. (2012). Lifetime Enhancement in Wireless Sensor Networks Using Fuzzy Approach and A-Star Algorithm. IEEE Sens. J..

[27] Huang Y., Martínez J.F., Díaz V.H., Sendra J. (2014). Localized and Energy-Efficient Topology Control in Wireless Sensor Networks Using Fuzzy-Logic Control Approaches. Math. Probl. Eng..

[28] Jiang H., Sun Y., Sun R., Xu H. (2013). Fuzzy-logic-based Energy Optimized Routing for Wireless Sensor Networks. Int. J. Distrib. Sens. Netw..

[29] Bhunia S.S., Das B., Mukherjee N. Multi criteria decision analysis assisted routing in wireless sensor network using fuzzy rules. Proceedings of the 2015 International Conference on Distributed Computing and Networking.

[30] Viittala H., Hamalainen M., Iinatti J. Zone-based fuzzy routing for WBANs. Proceedings of the International Symposium on Medical Information and Communication Technology.

[31] Jain S., Chawla M., Soares V.N.G.J., Rodrigues J.J. (2014). Enhanced fuzzy logic based spray and wait routing protocol for delay tolerant networks. Int. J. Commun. Syst..

[32] Jain R., Garg S. (2014). Dynamic source routing protocol for ad hoc network using the concept intelligent agent fuzzy logic. Int. J. Eng. Manag. Res..

[33] Singhal A., Daniel A.K. Fuzzy logic based stable on-demand multipath routing protocol for mobile Ad hoc network. Proceedings of the International Conference on Advanced Computing & Communication Technologies.

[34] Taheri H., Neamatollahi P., Younis O.M., Naghibzadeh S., Yaghmaee M.H. (2012). An energy-aware distributed clustering protocol in wireless sensor networks using fuzzy logic. Ad Hoc Netw..

[35] Rana S., Bahar A.N., Islam N., Islam J. (2015). Fuzzy based Energy efficient multiple cluster head selection routing protocol for wireless sensor networks. Int. J. Comput. Netw. Inf. Secur..

[36] Bagci H., Yazici A. (2013). An energy aware fuzzy approach to unequal clustering in wireless sensor networks. Appl. Soft Comput..

[37] Mao S., Zhao C., Zhou Z., Ye Y. (2013). An improved fuzzy unequal clustering algorithm for wireless sensor network. Mob. Netw. Appl..

[38] Jahanshahi M., Rahmani S., Ghaderi S. (2013). An efficient cluster head selection algorithm for wireless sensor network using fuzzy inference systems. Int. J. Smart Electr. Eng..

[39] Singh A.K., Purohit N., Varma S. (2013). Fuzzy logic based clustering in wireless sensor networks: A survey. Int. J. Electron..

[40] Jarupan B., Ekici E. (2011). A survey of cross-layer design for VANETs. Ad Hoc Netw..

[41] Kullkarni R.V., Forster A., Venayagamoorthy G.K. (2011). Computational Intelligence in Wireless Sensor Networks: A Survey. IEEE Commun. Surv. Tutor..

[42] Feng G. (2006). A Survey on Analysis and Design of Model-Based Fuzzy Control Systems. IEEE Trans. Fuzzy Syst..

